# Biocompatibility of Genipin and Glutaraldehyde Cross-Linked Chitosan Materials in the Anterior Chamber of the Eye

**DOI:** 10.3390/ijms130910970

**Published:** 2012-09-04

**Authors:** Jui-Yang Lai

**Affiliations:** 1 Institute of Biochemical and Biomedical Engineering, Chang Gung University, Taoyuan 33302, Taiwan; E-Mail: jylai@mail.cgu.edu.tw; Tel.: +886-3-211-8800 (ext. 3598); Fax: +886-3-211-8668; 2 Biomedical Engineering Research Center, Chang Gung University, Taoyuan 33302, Taiwan; 3 Molecular Medicine Research Center, Chang Gung University, Taoyuan 33302, Taiwan

**Keywords:** biocompatibility, chemical cross-linking, chitosan, ocular anterior chamber

## Abstract

Chitosan is a naturally occurring cationic polysaccharide and has attracted much attention in the past decade as an important ophthalmic biomaterial. We recently demonstrated that the genipin (GP) cross-linked chitosan is compatible with human retinal pigment epithelial cells. The present work aims to further investigate the *in vivo* biocompatibility of GP-treated chitosan (GP-chi group) by adopting the anterior chamber of a rabbit eye model. The glutaraldehyde (GTA) cross-linked samples (GTA-chi group) were used for comparison. The 7-mm-diameter membrane implants made from either non-cross-linked chitosan or chemically modified materials with a cross-linking degree of around 80% were inserted in the ocular anterior chamber for 24 weeks and characterized by slit-lamp and specular microscopic examinations, intraocular pressure measurements, and corneal thickness measurements. The interleukin-6 expressions at mRNA level were also detected by quantitative real-time reverse transcription polymerase chain reaction. Results of clinical observations showed that the overall ocular scores in the GTA-chi groups were relatively high. In contrast, the rabbits bearing GP-chi implants in the anterior chamber of the eye exhibited no signs of ocular inflammation. As compared to the non-cross-linked counterparts, the GP-chi samples improved the preservation of corneal endothelial cell density and possessed better anti-inflammatory activities, indicating the benefit action of the GP cross-linker. In summary, the intracameral tissue response to the chemically modified chitosan materials strongly depends on the selection of cross-linking agents.

## 1. Introduction

It is well known that biocompatibility is an important factor in determining the success of new medical implants and devices in the physiological environment. The word “biocompatibility” refers to the ability of a material to perform with an appropriate host response in a specific situation [1]. Although the eye has been considered to be an immune privileged site, the *in vivo* safety of a biomaterial should be checked before its ophthalmic application. Over the past few years, the anterior chamber of a rabbit eye model was used in our laboratory to test the ocular biocompatibility of various kinds of materials such as amniotic membrane [2], hyaluronic acid [3], gelatin [4], poly(2-hydroxyethyl methacrylate)-*co*-poly(acrylic acid) [5], and gelatin-*g*-poly(*N*-isopropylacrylamide) [6]. A methodology based on this animal model for biocompatibility assessment in an immune privileged site has several advantages, including convenient access to view, excellent tissue sensitivity, and reliable ophthalmic parameters [2].

Chitosan, a naturally occurring cationic polysaccharide, is obtained by the deacetylation of chitin. It is primarily composed of repeating d-glucosamine units and has wide biomedical applications, such as tissue engineering/regenerative medicine [7,8] and controlled drug/gene delivery [9,10]. Cross-linked chitosan has been proven to enhance structural and biological stability by joining molecules together [11]. Genipin (GP) is a cross-linker that can be isolated from the fruits of *Gardenia jasminoides* Ellis [12]. Because of its low cytotoxicity, GP has gained increasing interest in the field of biomaterial processing technology. In 2001, Mi *et al.* showed that cross-linking of chitosan membrane using GP reduced its tensile strain, swelling ratio, and enzymatic degradability [13]. A study from Chiono *et al.* reported that the GP cross-linked chitosan/gelatin blends with optimal composition were able to support neuroblastoma cell adhesion and proliferation [14]. Karnchanajindanun *et al.* also demonstrated that the controlled release of bovine serum albumin from GP cross-linked chitosan microspheres could be useful for the tailoring of a protein drug delivery system [15].

Although the ophthalmic application of GP treated chitosan is rarely found in the literature [16], the potential benefit of this naturally occurring cross-linker for intraocular surgery is evaluated. In 2006, the group of Kitano examined the effectiveness of herbal medicine (*i.e*., inchin-ko-to) in preventing posterior capsule opacification, and concluded that its main bioactive component, GP, may suppress α-TN4 lens cell fibrogenic behaviors [17]. Meanwhile, they also noted the inhibitory effect of GP on the injury-induced fibrogenic responses in subconjunctival fibroblasts following trabeculotomy [18]. Recently, Avila *et al.* showed that a GP cross-linking technique is useful to treat corneal ectasia and diseases involving corneal melting, due to a significant increase in biomechanical strength of collagenous tissue [19]. These findings together suggest the practical value of GP in clinical ophthalmology.

In light of the promising results, it may be possible to develop GP cross-linked biomaterials for ocular therapeutics, tissue repair, and pharmacology. This situation has motivated us to extend our previous work in exploring the *in vitro* responses of retinal pigment epithelial cells to chemically modified chitosan materials [20]. The aim of the present paper is to further investigate the *in vivo* biocompatibility of GP treated chitosan (GP-chi group) by adopting an appropriate animal model. The glutaraldehyde (GTA) cross-linked samples (GTA-chi group) were used for comparison on the effect of cross-linker type. The 7-mm-diameter membrane implants made from either non-cross-linked chitosan or counterparts with cross-linking degree of around 80% were inserted in the ocular anterior chamber. During the follow-up period of 24 weeks, the intracameral tissue reaction was analyzed by slit-lamp and specular microscopic examinations, intraocular pressure measurements, and corneal thickness measurements. The inflammatory response was also monitored by interleukin-6 (IL-6) expressions. To the best of our knowledge, this is the first report to assess the ocular biocompatibility of GP cross-linked chitosan materials in the anterior chamber of the eye.

## 2. Results

### 2.1. Biomicroscopic Examinations

**Figure 1:**
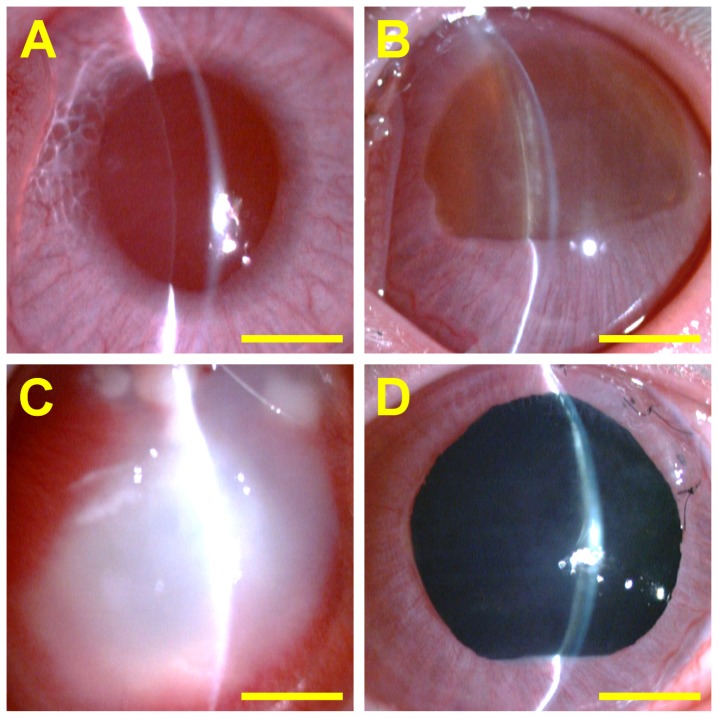
Representative slit-lamp biomicroscopic images of rabbit eyes 24 weeks after surgical insertion of various chitosan implants in the ocular anterior chamber. (**A**) Ctrl (sham-operated); (**B**) Chi; (**C**) GTA-chi; and (**D**) GP-chi groups. Scale bars: 3 mm.

Figure 1 shows representative slit-lamp biomicroscopic images for each group. After surgery for 24 weeks, the cornea in the sham-operated rabbits (received no implant) was clear. Additionally, the anterior chamber was quiet (no cells or flare). In the Chi groups, the 7-mm-diameter membranes were still visible at the graft site. However, the structural integrity of the implants was deteriorated since significant loss of non-cross-linked chitosan materials might have occurred during a 24-week *in vivo* degradation. The rabbit eyes showed very mild inflammation in the anterior chamber. After intraocular implantation of GTA-chi samples, severe ocular tissue responses, including aqueous flare, anterior chamber fibrin, corneal cloudiness, corneal neovascularization, iris neovascularization, and lens opacity were observed. By contrast, in the GP-chi groups, the implants appeared dark blue in color, mainly due to the formation of dark blue pigments via the reaction of GP with chitosan. These membranes remained stable in all rabbits at 24 weeks following surgery and demonstrated a slight reduction in size by 5%–10%. Of note, the eyes bearing the GP cross-linked materials showed no evidence of ocular inflammation.

**Figure 2:**
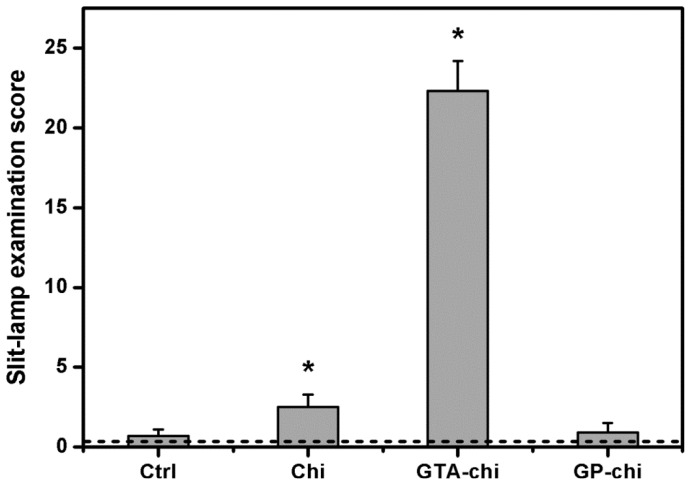
Slit-lamp examination scores of rabbit eyes 24 weeks after surgical insertion of various chitosan implants in the ocular anterior chamber. The dash line represents the preoperative score. An asterisk indicates statistically significant differences (* *p* < 0.05; *n* = 6) as compared to the Ctrl (sham-operated) groups.

The slit-lamp examination score for each group is shown in Figure 2. Before surgery, the overall ocular scores were relatively low, ranging from 0.2 to 0.5. In the Ctrl groups, the total scores were 0.7 ± 0.4, which indicates that the inflammation manifestations were not found in the sham-operated rabbits at 24 weeks post operation. After exposure to the Chi materials, the animals had a score of 2.5 ± 0.8, suggesting very little inflammation. Our data demonstrated that among all the test samples studied, the GTA-chi implants might induce severe inflammatory responses, leading to significantly higher scores. On the other hand, there were no significant differences between Ctrl and GP-chi groups (*p* > 0.05) with respect to aqueous flare, anterior chamber fibrin, corneal cloudiness, corneal neovascularization, iris neovascularization, and lens opacity measurements.

**Figure 3:**
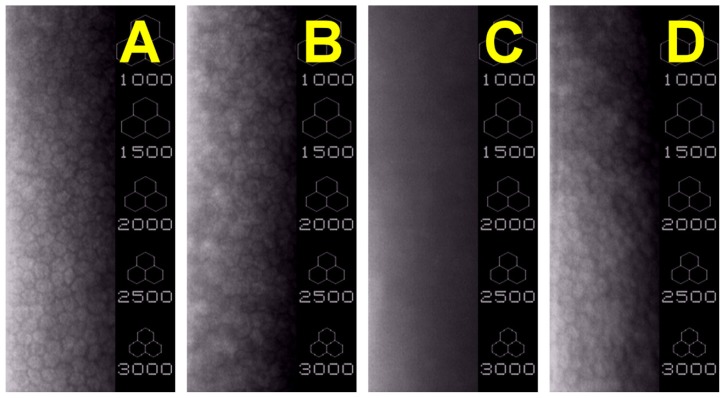
Typical specular microscopic images of rabbit corneal endothelium 24 weeks after surgical insertion of various chitosan implants in the ocular anterior chamber. (**A**) Ctrl (sham-operated); (**B**) Chi; (**C**) GTA-chi; and (**D**) GP-chi groups.

The morphology of corneal endothelial cells in rabbit eyes was observed by specular microscopy. As shown in Figure 3A, the cells on Descemet’s membrane packed together and exhibited a typical hexagonal shape, indicating that the sham operation does not alter corneal endothelial morphology. After surgical insertion of Chi implants in the ocular anterior chamber, the rabbit corneal endothelium showed some irregular shape of cells (Figure 3B). In the GTA-chi groups, the cellular hexagonality could not be identified (Figure 3C). For the animals bearing the GP cross-linked materials, a single continuous monolayer of cells was present on the posterior surfaces of cornea (Figure 3D). In addition, the individual corneal endothelial cells had distinct borders and possessed similar morphological features to those in the Chi-implanted eyes.

**Figure 4:**
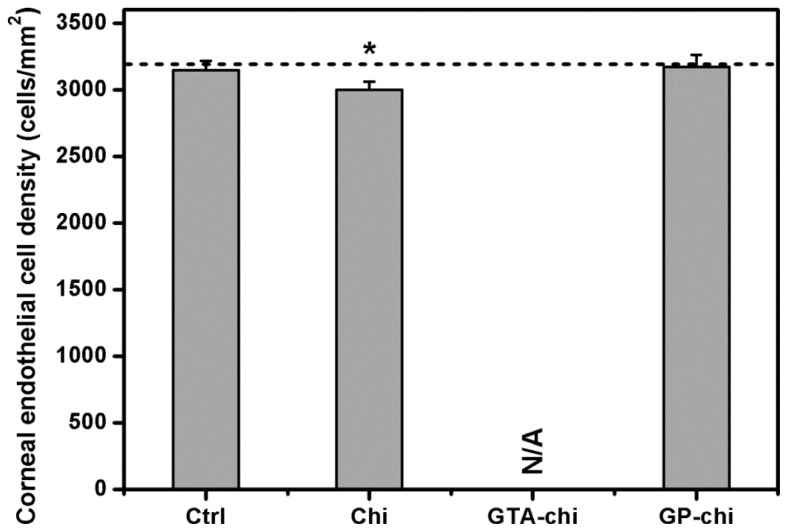
Specular microscopy measurements of corneal endothelial cell density 24 weeks after surgical insertion of various chitosan implants in the ocular anterior chamber. The dash line represents the preoperative cell density. An asterisk indicates statistically significant differences (* *p* < 0.05; *n* = 6) as compared to the Ctrl (sham-operated) groups. N/A: Not applicable, because the biological responses are too severe to be observed.

Figure 4 shows the results of quantitative specular microscopic analysis of rabbit corneal endothelium. At pre-operation, the mean corneal endothelial cell density was approximately 3200 cells/mm^2^. No significant difference was noted in the cell density of Ctrl groups (3145 ± 71 cells/mm^2^) compared with the values before surgery (*p* > 0.05). After exposure to the Chi implants for 24 weeks, the animals had a significantly lower cell density (2998 ± 63 cells/mm^2^). Because the biological responses were too severe to be observed, the endothelial cell counts of GTA-chi groups were not available from specular microscopic images. The cell density did not show a significant difference between the sham-operated and GP-chi groups (3172 ± 90 cells/mm^2^; *p* > 0.05).

### 2.2. Intraocular Pressure Measurements

**Figure 5:**
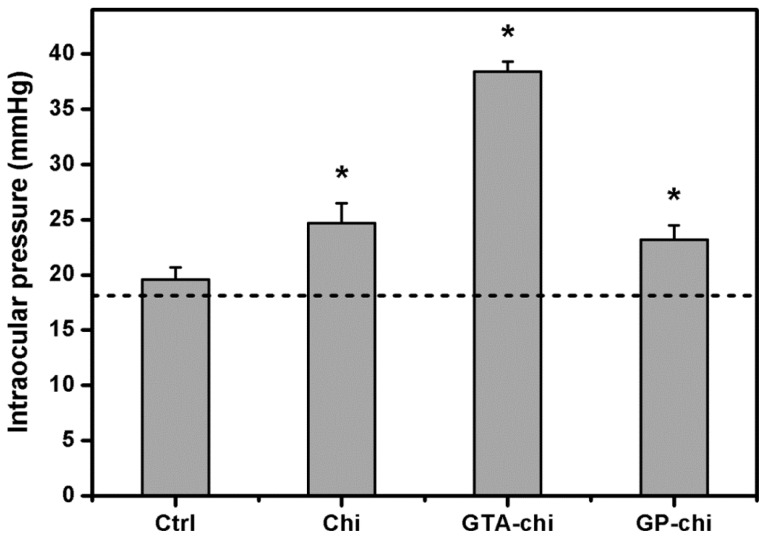
Measurements of intraocular pressure 24 weeks after surgical insertion of various chitosan implants in the ocular anterior chamber. The dash line represents the preoperative intraocular pressure. An asterisk indicates statistically significant differences (* *p* < 0.05; *n* = 6) as compared to the Ctrl (sham-operated) groups.

The IOP profile of the four groups is shown in Figure 5. The sham-operated eyes had an IOP of 19.6 ± 1.1 mmHg, which was not significantly different from that of normal rabbit eyes. The mean IOP values were increased by surgical insertion of various chitosan implants in the ocular anterior chamber. At 24 weeks post-operation, the IOP in the Chi, GTA-chi, and GP-chi groups was 24.7 ± 1.8, 38.4 ± 0.9, and 23.2 ± 1.3 mmHg, respectively.

### 2.3. Corneal Thickness Measurements

**Figure 6:**
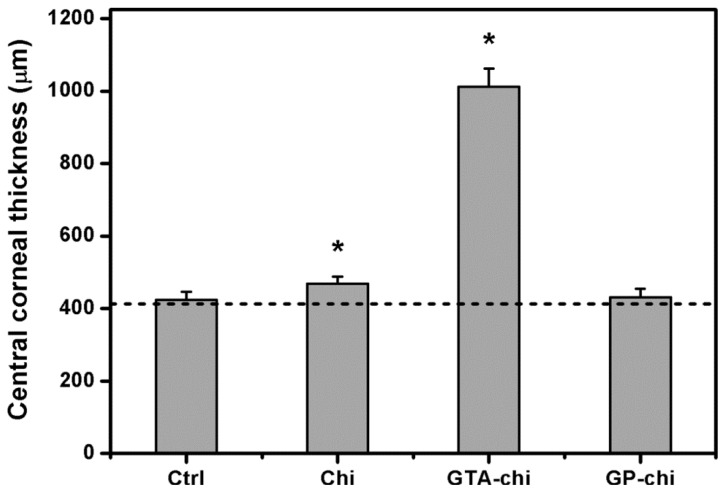
Measurements of central corneal thickness 24 weeks after surgical insertion of various chitosan implants in the ocular anterior chamber. The dash line represents the preoperative corneal thickness. An asterisk indicates statistically significant differences (* *p* < 0.05; *n* = 6) as compared to the Ctrl (sham-operated) groups.

The CCT changes of the surgical corneas of the four groups are shown in Figure 6. Mean preoperative thickness values ranged from 411.3 to 426.5 μm. The CCT of the sham-operated rabbit eyes (423.6 ± 22.1 μm) and the GP-chi-implanted eyes (430.2 ± 24.7 μm) was within the normal range. By contrast, the thickness value in the Chi groups was 469.1 ± 18.5 μm, which was significantly higher than that in the Ctrl groups (*p* < 0.05). It was noted that the CCT was increased to a value larger than 1000 μm after implantation of GTA-chi samples in the ocular anterior chamber for 24 weeks.

### 2.4. Quantitative Real-Time Reverse Transcription Polymerase Chain Reaction Analyses

**Figure 7:**
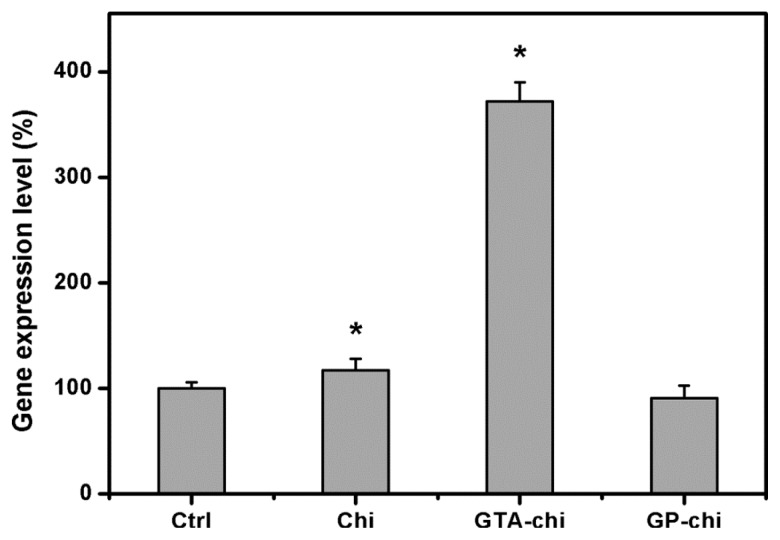
Gene expression levels of interleukin-6 in rabbit corneal endothelium exposed to various chitosan implants for 24 weeks by real-time reverse transcription polymerase chain reaction. Normalization is done by using glyceraldehyde-3-phosphate dehydrogenase. Data in the experimental groups are percentages relative to that of Ctrl (sham-operated) groups. An asterisk indicates statistically significant differences (* *p* < 0.05; *n* = 6) as compared to the Ctrl (sham-operated) groups.

Figure 7 shows the gene expression levels of IL-6 in rabbit corneal endothelium exposed to various chitosan implants for 24 weeks. In this study, by using quantitative real-time RT-PCR, the detected expression level for both genes in the Ctrl groups was defined as 100%. The IL-6 mRNA expression in the Chi, GTA-chi, and GP-chi groups was 117.2 ± 10.6, 371.8 ± 18.2, and 90.7% ± 12.0%, respectively, which demonstrated significant up-regulation of IL-6 in the animals bearing the GTA cross-linked materials.

## 3. Experimental Section

### 3.1. Materials

Chitosan (Cat. No. 50494), derived from crab shell, is a commercial powder supplied by Fluka (Milwaukee, WI, USA). According to information from the manufacturer, the chitosan samples used as raw materials had a degree of deacetylation of 95%–98% and a molecular weight of approximately 150 kDa. Genipin was purchased from Wako Pure Chemical Industries (Osaka, Japan). Glutaraldehyde was obtained from Sigma-Aldrich (St. Louis, MO, USA). Phosphate-buffered saline (PBS, pH 7.4) was purchased from Biochrom AG (Berlin, Germany). TRIzol reagent was obtained from Gibco-BRL (Grand Island, NY, USA). Deionized water used was purified with a Milli-Q system (Millipore, Bedford, MA, USA). All the other chemicals were of reagent grade and used as received without further purification.

### 3.2. Preparation of GP or GTA Cross-Linked Chitosan Membranes

The chitosan membranes (Chi group) were prepared by the solution casting method. In brief, chitosan (1 g) was added to 1% *v*/*v* aqueous acetic acid (50 mL) with stirring until complete dissolution. To remove insoluble substances, the solution was passed through a filter paper (Tokyo Roshi Kaisha, Tokyo, Japan). Then, 0.5 mL of chitosan solution was poured into a well of a 24-well plate (Falcon, Becton Dickinson Labware, Franklin Lakes, NJ, USA) and air-dried for 2 days at 25 °C to obtain membranes (about 10 μm thick). The membrane samples were incubated in a 0.5 N NaOH solution for 1 h and washed extensively with deionized water until neutrality.

The chitosan materials were treated with GTA (GTA-chi group) or GP (GP-chi group) by respectively immersing the membrane samples in 5 mL of PBS containing 10 mM cross-linker. The cross-linking reaction was allowed to proceed at 25 °C for different time periods. To eliminate the residual GTA or GP, the cross-linked membranes were washed extensively in deionized water and cut out using a 7-mm-diameter corneal trephine device. Then, the samples were dried *in vacuo* for 24 h and sterilized in a graded series of ethanol solutions and thoroughly rinsed in sterilized PBS for use in the *in vivo* experiments.

The amount of free amino groups of chitosan membranes was determined to evaluate their extent of cross-linking. The test sample was weighed and heated with a ninhydrin solution for 20 min. After the test solution was cooled to room temperature and diluted in 95% ethanol, the optical absorbance of the solution was recorded with a UV-visible spectrophotometer (Thermo Scientific, Waltham, MA, USA) at 570 nm using glycine at various known concentrations as standard [21,22]. The amount of free amino groups in the chitosan materials before (*C*_b_) and after (*C*_a_) cross-linking is proportional to the optical absorbance of the solution. The extent of cross-linking of the chitosan membranes was calculated as cross-linking index (%) = ((*C*_b_ − *C*_a_)/*C*_b_) × 100. Results were averaged over five independent runs. In this study, the GTA and GP treated implants with the same extent of cross-linking (*i.e*., 76.8% ± 2.6% for GTA-chi and 78.5% ± 2.8% for GP-chi) were characterized by various *in vivo* assays.

### 3.3. Animals

All animal procedures were approved by the Institutional Review Board and were performed in accordance with the ARVO (Association for Research in Vision and Ophthalmology) Statement for the Use of Animals in Ophthalmic and Vision Research. Twenty-four adult New Zealand white rabbits (National Laboratory Animal Breeding and Research Center), weighing from 3.0 to 3.5 kg and 16–20 weeks of age, were used for this study. Animals were healthy and free of clinically observable ocular surface disease. A surgical operation was performed on a single eye of each animal, with no procedure being performed on the other eye. In the three test groups (Chi, GTA-chi and GP-chi) of animals (six rabbits/group), the chitosan implants were inserted in the anterior chamber of the eye. The remaining six rabbits received no implant (only corneal/limbal incision) and served as a control group (Ctrl).

### 3.4. Surgery

The rabbits were anesthetized intramuscularly with 2.5 mg/kg body weight of tiletamine hydrochloride/zolazepam hydrochloride mixture (Zoletil; Virbac, Carros, France) and 1 mg/kg body weight of xylazine hydrochloride (Rompun; Bayer, Leverkusen, Germany), and topically with two drops of 0.5% proparacaine hydrochloride ophthalmic solution (Alcaine; Alcon-Couvreur, Puurs, Belgium). After disinfection and sterile draping of the operation site, the pupil was dilated with one drop of 1% atropine sulfate ophthalmic solution (Oasis, Taipei, Taiwan, ROC), and a lid speculum was put in place. Under the surgical microscope (Carl Zeiss, Oberkochen, Germany), the cornea was penetrated near the limbus by using a slit knife. Then, the corneal/limbal incision was enlarged to 7.5 mm with corneal scissors to allow the insertion of an implant in the anterior chamber. The incision site was finally closed with 10–0 nylon sutures.

### 3.5. Biomicroscopic Examinations

To determine the implant-tissue interaction in the anterior chamber, the rabbits were anesthetized under the same conditions as for surgery. Ophthalmic evaluations were performed before and 24 weeks after surgical insertion of material implants. The morphology of anterior segment of the eye including corneal and lens clarity, the degree of anterior chamber activity, iris, and implants was observed by slit-lamp biomicroscopy (Topcon Optical, Tokyo, Japan).

The ocular grading method used for biomicroscopic examinations is shown in Table 1. During clinical assessment, six parameters were recorded from rabbit eyes and were numerically graded on an increasing severity scale of 0–4. The means of the ocular scores for each parameter were quantitatively calculated to be the sum of the scores for each group, divided by the total number of eyes in that group. Total score was expressed as summary of six mean ocular scores for each group.

The corneal endothelial cell density in rabbit eyes was measured by specular microscopy (Topcon Optical). Each data point is an average of three independent observations.

### 3.6. Intraocular Pressure Measurements

The intraocular pressure (IOP) was measured using a Schiotz tonometer (AMANN Ophthalmic Instruments, Liptingen, Germany), calibrated according to the manufacturer’s instructions. For each IOP determination, five readings were taken on each eye, and the mean was calculated.

### 3.7. Corneal Thickness Measurements

Central corneal thickness (CCT) was determined using an ultrasonic pachymeter (DGH Technology, Exton, PA, USA) with a hand-held solid probe. During the measurements, the probe tip of the pachymeter was held perpendicular on the central cornea. An average of ten readings was taken.

### 3.8. Quantitative Real-Time Reverse Transcription Polymerase Chain Reaction Analyses

**Figure 8:**
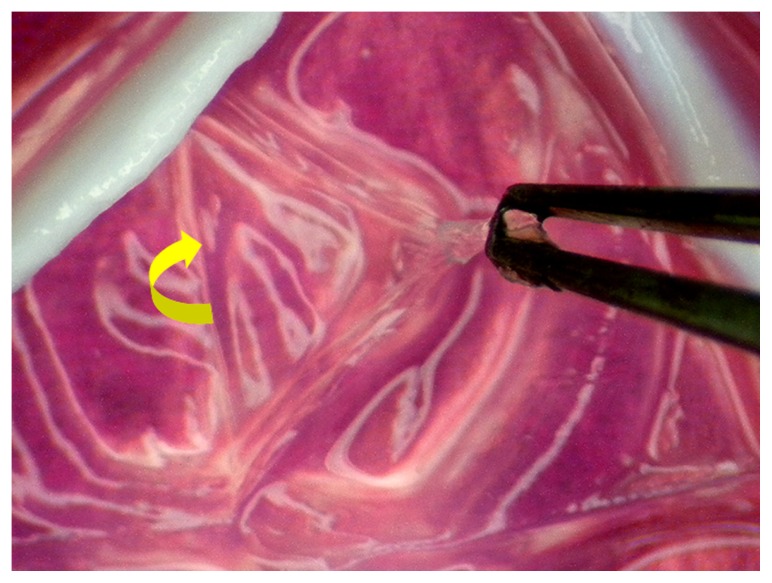
Tissue procurement for *in vivo* real-time reverse transcription polymerase chain reaction studies. An asterisk indicates the direction in which the Descemet’s membrane with the attached endothelium is stripped from the corneal stroma.

At the end of experiments, the animals were euthanized with CO_2_ gas. The excised rabbit corneas were then processed for quantitative real-time reverse transcription polymerase chain reaction (RT-PCR) analyses. Under a dissecting microscope (Leica, Wetzlar, Germany), the Descemet’s membrane with the attached endothelium was aseptically stripped from the corneal stroma and washed three times with PBS (Figure 8). For *in vivo* real-time RT-PCR, total RNA was isolated from corneal endothelium with TRIzol reagent according to the manufacturer’s procedure. Reverse transcription of the extracted RNA (1 μg) was performed using ImProm-II (Promega, Madison, WI, USA) and Oligo(dT)_15_ primers (Promega). The sequences of the primer pairs for each gene are listed in Table 2. Quantitative real-time RT-PCR was performed on a Light-Cycler instrument (Roche Diagnostics, Indianapolis, IN, USA) according to the manufacturer’s instructions with FastStart DNA Master SYBR Green I reagent (Roche Diagnostics). Each sample was determined in six replicates, and the gene expression results were normalized to the expression of glyceraldehyde-3-phosphate dehydrogenase (GAPDH).

### 3.9. Statistics

Results were expressed as mean ± standard deviation. Comparative studies of means were performed using one-way analysis of variance (ANOVA). Significance was accepted with *p* < 0.05.

## 4. Discussion

Biocompatibility is a prerequisite for the development of potential ophthalmic biomaterials. It has been recognized that biocompatibility is controlled mainly by the interface between foreign materials and host living cells/tissues [23]. In our previous study, the cellular responses to various chitosan materials are examined using an *in vitro* model based on ARPE-19 cell line culture [20]. The results indicate that the cells exposed to GTA treated chitosan membranes may have significantly higher cytotoxicity, IL-6 levels, and number of apoptotic cells than did those exposed to GP cross-linked samples. Here, we investigated the ocular biocompatibility of chemically cross-linked chitosan implants by adopting the anterior chamber of the rabbit eye model.

Ophthalmic examinations including slit-lamp microscopy and specular microscopy were performed in all eyes to evaluate the status of the anterior segment. In the Ctrl groups, the sham operation (*i.e*., only corneal/limbal incision) does not cause signs of ocular inflammation and change in corneal endothelial cell morphology and density at 24 weeks post-operation. This is probably due to the fact that the initial responses to implantation of biomaterials are the acute and subacute phases of inflammation induced by surgical trauma [24]. After placement of material samples in the center of the anterior chamber, the implant-tissue interaction was monitored for 24 weeks. Our data showed that there is an extremely large amount of remnants of non-cross-linked chitosan membrane (approximately 60% of the original size) within the implantation region. Chitosan has a very slow degradation rate. Lu *et al.* have demonstrated that the chitosan films exhibit almost no degradation during 8 weeks of *in vitro* incubation in lysozyme solution [25]. The results of the study suggest that the chemical cross-linking may increase resistance to *in vivo* degradation. Mi *et al.* have reported that while the intramuscularly injected fresh chitosan microspheres retrieved at 12 weeks post-operation are already degraded into fragments, the degradation of the GP cross-linked chitosan counterparts is not significant after 20 weeks of implantation [26]. Our present results are compatible with their findings and suggest that the GP treated chitosan implants with cross-linking degree of around 80% exhibit relatively little biodegradation in a unique ocular immune privileged site. It is of interest to note that for all test materials, the lower part of the implants disappears. In terms of aqueous humor circulation, the tissue fluid leaves the eye by passing through the trabecular meshwork into the canal of Schlemm [27]. One possible explanation is that the elimination of degraded chitosan molecules from the eye is highly correlated with the aqueous humor drainage.

The *in vitro* ocular biocompatibility of non-cross-linked chitosan membranes is demonstrated by the absence of any signs of toxicity or inflammation reaction [20]. However, the present work reports that the rabbit eyes exposed to the implants made from the same material may have very mild inflammation in the ocular anterior chamber. The differences in the obtained results can be attributed to different testing models. Although the *in vitro* cell culture models have fewer inherent variations than the corresponding *in vivo* animal model, the *in vitro* toxicological studies are usually aimed at investigating the short-term effects of materials on acute toxicity. In our laboratory, an indirect contact methodology has also been used to evaluate cytotoxicity of biopolymers such as gelatin [28] and hyaluronic acid [29] by a 2-day incubation of corneal endothelial cells with test hydrogels. In comparison to the *in vitro* experiments, the *in vivo* tests can more accurately determine the material biocompatibility, but they are usually more expensive and elaborate. During 24 weeks of follow-up evaluations, the chitosan implants were directly in contact with the tissues of the anterior segment of the eye. The continued residence of material samples in the intraocular cavity may elicit a much greater response.

In the GTA-chi groups, the implants induce undesirable host reactions and some adverse biological effects, suggesting that the GTA cross-linking produces a strong influence on the interaction between chitosan materials and ocular tissues. Given that the GTA has very reactive properties and is easily interacted with cell surface, such a cross-linker can result in cytotoxicity and apoptosis [4]. Our findings indicate that the GTA-chi samples are less biocompatible than their GP-chi counterparts. It is noteworthy that the chitosan materials cross-linked with GP are well tolerated without causing intraocular inflammation. The anti-inflammatory activities of GP have attracted much attention over the past few years. Koo *et al.* have demonstrated that GP exhibits inhibitory effect on nitric oxide production through the inhibition of nuclear factor-κB activation [30]. Nam *et al.* have also reported that the treatment of GP to lipopolysaccharide-stimulated microglia is effective at decreasing nitric oxide release, and suggested that the aglycon of geniposide can inhibit microglial activation in a mouse model of brain inflammation [31]. More recently, Jeon *et al.* have shown that GP up-regulates heme oxygenase-1 via PI3-kinase-JNK1/2-Nrf2 signaling pathway to enhance the anti-inflammatory capacity in RAW264.7 macrophages [32].

The corneal endothelium is a thin cell monolayer that forms the posterior boundary of the cornea and maintains corneal clarity [33]. Cellular hexagonality is a sensitive indicator of corneal endothelial damage. We have previously demonstrated that the corneal endothelium surrounding the GTA treated gelatin implants exhibits significantly lower percent hexagonality than did those of rabbits bearing the carbodiimide cross-linked gelatin hydrogels [4]. In addition, scanning electron microscopy of corneal biopsy does not reveal any endothelial morphological abnormalities, indicating good biocompatibility of carbodiimide modified hyaluronic acid implants [3]. In the present study, the corneal endothelium is investigated with specular microscopy, which is a noninvasive test capable of imaging corneal endothelial cells *in vivo*. The effects of biomaterial implants on the endothelial cell morphology and count are also evaluated in relation to cross-linking agent type. The exposure of rabbit eyes to the GTA cross-linked chitosan implants causes severe tissue responses, whereas no adverse inflammatory reaction is noted after contact with GP treated chitosan samples. Although the endothelial cell count was not statistically different between the Ctrl and GP-chi groups, some irregularly shaped corneal endothelial cells and tiny dark areas were noted in Figure 3D. One possible explanation is that the GP cross-linked chitosan implants can float freely in the aqueous humor of the anterior chamber to touch corneal endothelium, and thereby cause mechanical damage to the tissue.

It is known that the IOP is an important measure of the balance between aqueous humor production and drainage. The disturbance of aqueous humor circulation is observed when the biomaterial implants are placed in the ocular anterior chamber [24]. Our previous work has shown that the biodegradation of hydrogel carriers has a significant impact on the control of IOP and the outcome of corneal cell sheet-based therapy [34]. Although the *in vivo* presence of chitosan materials may increase aqueous humor outflow resistance in the trabecular meshwork, the rabbits bearing the GTA cross-linked chitosan implants reveal additional elevated IOP. These findings imply poor biocompatibility of GTA-chi samples, which corroborates biomicroscopic data. On the other hand, given that the corneal endothelial cells maintain corneal hydration and transparency by a pump-leak mechanism, CCT is considered as an indicator associated with corneal diseases [35]. The increase in CCT usually reflects the alteration of corneal endothelial cell functions. The results of clinical observations indicate that at 24 weeks post-operatively, the GTA-chi-implanted eyes develop marked corneal edema with considerable endothelial damage. By contrast, the GP-chi implants show good ocular tolerability in the anterior chamber of the rabbit eye model.

Naturally occurring polymeric materials, including collagen [36], gelatin [37], hyaluronic acid [38], and chondroitin sulfate [39] have been demonstrated to be suitable for ocular tissue engineering applications. Chitosan is also an important biopolymer that has antibacterial and wound healing activities. To facilitate the understanding of their safety and performance in ophthalmic fields, the chitosan samples modified with cross-linkers must be evaluated by determinations of the tissue/biomaterial interactions. Quantitative real-time RT-PCR is the most sensitive technique for measuring gene expressions of IL-6 in rabbit corneal endothelium exposed to various chitosan implants for 24 weeks. Our present results indicate that while the GTA-chi samples up-regulate IL-6 gene levels, the GP-chi counterparts do not promote inflammation. It has been reported that the GTA can react with free amino groups of chitosan to form Michael-type adducts with terminal aldehydes, thereby causing harmful irritation to cultured ARPE-19 cells [20]. This may explain the findings of the current study.

## 5. Conclusion

In conclusion, using the anterior chamber of a rabbit eye model, we found that the intracameral tissue response to the chemically modified chitosan materials strongly depended on the selection of cross-linker. While satisfactory *in vivo* biocompatibility of GP-chi implants was shown at post-operative 24 weeks, the GTA-chi implants caused significant intraocular inflammation. The applications of GP cross-linked chitosan nanoparticles for drug delivery in the treatment of posterior segment diseases are currently being investigated.

## Figures and Tables

**Table 1:** Ocular grading system used for biomicroscopic examinations.

Parameter	Ocular score				

	0	1	2	3	4
Aqueous flare	Normal	Mild	Moderate	Severe	N/A a
Anterior chamber fibrin	None	Mild	Moderate	Severe	N/A a
Corneal cloudiness severity	Normal	Mild	Moderate	Severe	N/A a
Corneal neovascularization	None	Mild	<180°	180°–360°	N/A a
Iris neovascularization	None	Mild	<180°	180°–360°	N/A a
Lens opacity	None	Mild	Moderate	Severe	N/A a

a Not applicable, because the biological responses are too severe to be observed.

**Table 2:** Sequences of primers used in gene expression analyses.

Genes a	Forward (5′-3′)	Reverse (5′-3′)
IL-6	AAGAAAACACCAGGGTCAGCAT	CTTGAGGGTGGCTTCTTCATTC
GAPDH	TTGCCCTCAATGACCACTTTG	TTACTCCTTGGAGGCCATGTG

a IL-6: interleukin-6; GAPDH: glyceraldehyde-3-phosphate dehydrogenase.
